# Supplementary figures and images for: Effect of bed height on laryngoscopy force and operator ergonomics during simulated endotracheal intubation: A randomized controlled study
Source: PLoS One. 2025 Oct 10;20(10):e0333104. doi: 10.1371/journal.pone.0333104 (PMC12513669; doi:10.1371/journal.pone.0333104)

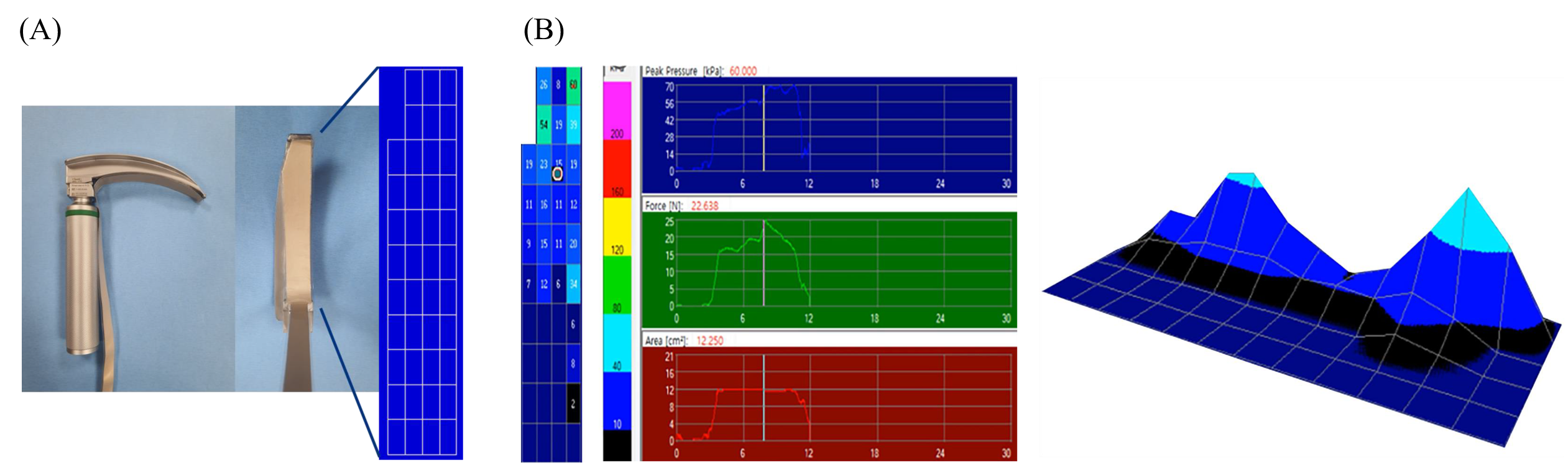

Supplement: S1 Fig — (A) Pliance® pressure sensor applied to the contact surface of Macintosh 3 blade. (B) Force data capture process and real-time display of pressure and force with visual assistance. (TIF) [file pone.0333104.s001.tif]
