# Supplementary material for: Effect of bed height on laryngoscopy force and operator ergonomics during simulated endotracheal intubation: A randomized controlled study
Source: PLoS One. 2025 Oct 10;20(10):e0333104. doi: 10.1371/journal.pone.0333104 (PMC12513669; doi:10.1371/journal.pone.0333104)
Supplement: S1 Table — SE standard error, CI confidence interval. aCarryover effect evaluated from GEE model with missing data not treated with imputation nor weighting Carryover effect was not found in other datasets used in sensitivity analysis. (DOCX) [file pone.0333104.s002.docx]

**Supplementary Table 1** Carryover, sequence, and period effects

|  | Carryover effect (1^st^ vs. 2^nd^ intubation) | Sequence effect (AX vs. XA sequence) | | | Period effect (Period 1 vs. 2) | | |
| --- | --- | --- | --- | --- | --- | --- | --- |
|  | *p* value^a^ | Estimate (SE) | 95% CI | *p* value | Estimate (SE) | 95% CI | *p* value |
| Peak force | 0.709 | 4.13 (1.83) | 0.54, 7.71 | 0.024 | 2.33 (1.22) | -0.05, 4.71 | 0.055 |
| Average force | 0.355 | 3.00 (0.92) | 1.19, 4.81 | 0.001 | -0.10 (0.63) | -1.34, 1.13 | 0.872 |
| Impulse force | 0.936 | 48.91 (16.85) | 15.89, 81.93 | 0.004 | 40.18 (13.13) | 14.44, 65.92 | 0.002 |

*SE* standard error, *CI* confidence interval. ^a^Carryover effect evaluated from GEE model with missing data not treated with imputation nor weighting Carryover effect was not found in other datasets used in sensitivity analysis
